# In shoulder adhesive capsulitis, ultrasound-guided anterior hydrodilatation in rotator interval is more effective than posterior approach: a randomized controlled study

**DOI:** 10.1007/s10067-020-05131-2

**Published:** 2020-05-08

**Authors:** Basant Elnady, Elsayed M. Rageh, Manal Shawky Hussein, Mohammed Hassan Abu-Zaid, Dalia El-Sayed Desouky, Tohamy Ekhouly, Johannes J. Rasker

**Affiliations:** 1 Department of Rheumatology, Rehabilitation and Physical Medicine, Benha University, Benha, Egypt; 2 Department of Rheumatology, Rehabilitation and Physical Medicine, Tanta University, Tanta, Egypt; 3 Department of public health and community medicine, Menoufia University, Shibin Al Kawm, Egypt; 4 Department of Radiology, Benha University, Benha, Egypt; 5 Faculty of Behavioral, Management and Social sciences, Department Psychology, Health and Technology, University of Twente, PO box 217, 7500 AE Enschede, Netherlands

**Keywords:** Adhesive capsulitis, Frozen shoulder, Rotator interval, Ultrasound-guided hydrodilatation

## Abstract

**Abstract:**

Shoulder adhesive capsulitis, also called frozen shoulder, is a musculoskeletal disorder associated with pain and functional disability. This study aimed to compare the effectiveness of shoulder ultrasound-guided hydrodilatation with corticosteroid, via rotator interval (RI) anteriorly, versus posterior approach, in adhesive capsulitis patients. All patients received exercise program following injection.

**Patients and methods:**

A prospective randomized controlled study among 60 consecutive adhesive capsulitis patients was randomized into two equal groups. Group I received ultrasound-guided hydrodilatation with corticosteroid, saline, and local anesthetic via posterior intra-articular approach; group II received the same ultrasound-guided hydrodilatation via anterior rotator interval approach. Both groups received guided stretching exercises for 3 months after injection. Baseline and 3 months evaluation of pain by visual analogue scale (VAS), shoulder pain and disability index (SPADI), and range of motion (ROM) had been recorded for all patients.

**Results:**

Both groups showed significant improvement 3 months after hydrodilatation regarding VAS pain, external rotation, and SPADI. Only in group II (RI anterior approach) improvement was observed regarding flexion and abduction. There was no improvement regarding extension or internal rotation in either group. When comparing the improvement in both groups after hydrodilatation, group II (anterior approach) showed a statistically significant higher level of improvement regarding VAS pain (*p* = 0.003), SPADI, flexion, abduction, and external rotation, compared to group I (*p* < 0.001). Extension, internal rotation, and adduction were not different.

**Conclusions:**

Ultrasound-guided anterior rotator interval hydrodilatation for adhesive capsulitis, followed by guided exercise, is clinically and functionally more effective than the conventional posterior approach.

**Table:** 

**Key Points** *• Ultrasound-guided hydrodilatation (with prednisolone acetate (40 mg), 1 ml of 2% lidocaine, and 15 ml saline) for adhesive capsulitis followed by guided exercise is clinically and functionally effective.* *• The ultrasound-guided anterior rotator interval approach is clinically and functionally significantly more effective than the conventional intra-articular posterior approach as it targets mainly the area of pathology.* *• This is the first prospective study comparing the effect of the anterior rotator interval approach versus the posterior approach in ultrasound-guided hydrodilatation in adhesive capsulitis patients.*	

## Introduction

Adhesive capsulitis and frozen shoulder syndrome are two terms that have been used to describe a painful and stiff shoulder [1]. The definition of adhesive capsulitis according to the American Shoulder and Elbow Surgeons is “a condition of uncertain etiology characterized by significant restriction of both active and passive shoulder motion that occurs in the absence of a known intrinsic shoulder disorder” [1].

Adhesive capsulitis is a common, but poorly understood, musculoskeletal disorder, with a prevalence of 2–5% in the general population [2, 3]. It is one of the most common disorders presenting to orthopedic surgeons [3]. Risk factors include trauma, diabetes mellitus, prolonged immobilization, autoimmune disorders, stroke, and myocardial infarcts [4].

Frozen shoulder usually is seen in the sixth decade of life, and onset is very uncommon before the age of 40. The peak age is 56, and the condition occurs slightly more frequently in women than men. In 6–17% of patients, also the other shoulder becomes affected, generally within 5 years, and after the first has resolved [4]. Capsulitis rarely occurs simultaneously bilaterally [5].

Progressive shoulder pain with gradual loss of passive and active range of motion (ROM), occurring in adhesive capsulitis, is caused by inflammation of the synovial lining capsule and generalized contracture of the glenohumeral joint [4].

The rotator interval is a space between the subscapularis and supraspinatus tendons; it contains the long head of the biceps tendon, the coracohumeral and the superior glenohumeral ligaments, and parts of the joint capsule. The rotator interval is important for keeping stability of the long head of biceps tendon and glenohumeral joint [6]. The anterior capsule and rotator interval are primarily involved in adhesive capsulitis. On MRI arthrography, patients with frozen shoulder had a significantly thickened coracohumeral ligament and a thickened joint capsule in the rotator cuff interval compared to controls and synovitis-like abnormalities at the superior border of the subscapularis tendon that were also significantly more common [7].

There are a lot of treatment options for adhesive capsulitis including rest, nonsteroidal anti-inflammatory drugs (NSAIDs), physiotherapy, and dynamic splinting [8].

Local intra-articular corticosteroid injection can be applied in conjunction with oral NSAIDs or oral corticosteroids in treating adhesive capsulitis, a method that was found to cause rapid pain relief that lasts for 6 weeks [9]. Hydrodilatation is an effective therapeutic intervention giving rapid symptomatic relief from adhesive capsulitis; this technique consists of an injection of a saline or a saline combined with corticosteroids that distend the capsule by hydrostatic pressure [10]. Hydrodilatation (also called hydrodistension) of the glenohumeral joint with normal saline and corticosteroid was found to decrease the intra-articular pressure and increase the shoulder volume capacity [10]. That is why the capsular distension was used for treatment of frozen shoulder, due to the physiological benefits of distending the contracted shoulder joints [10]. Hydrodilatation can be performed with fluoroscopic guidance or with ultrasound guidance, and both methods have similar outcomes. However, ultrasound-guided hydrodilatation has the benefit of avoiding the usage of ionizing radiation [11]. It is also quicker and cheaper and allows the assessment of the rotator cuff muscles [11].

Hydrodilatation had better results in management of adhesive capsulitis than manipulation under anesthesia [12]. A Cochrane review studied the safety and efficacy of hydrodilatation based on five trials (*n* = 196), with only one with low risk of bias, which demonstrated that distension with saline and corticosteroid was better than placebo for pain, function, and range of movement at 3 weeks [13]. This benefit was only maintained at 6 and 12 weeks for one of two scores measuring function. A second study, with high risk of bias, found that after 8 weeks, pain had improved compared to physical therapy alone. Three further trials, all with high risk of bias, reported conflicting, variable effects of arthrographic distension with corticosteroid, compared to distension alone, and arthrographic distension with corticosteroid compared to intra-articular corticosteroid injection. The authors conclude that there is “silver” level evidence that arthrographic distension with saline and corticosteroid provides short-term benefits in pain, range of movement, and function in adhesive capsulitis. It is uncertain whether this is better than alternative interventions [9].

The aims of the current study were:

- To compare the effectiveness of anterior ultrasound-guided hydrodilatation, via the rotator interval, versus posterior intra-articular ultrasound-guided hydrodilatation (with saline, corticosteroid, and lidocaine) in primary adhesive capsulitis, followed in both groups by a guided stretching exercise program
- To assess the outcome of pain, functional status, and range of motion in both groups and to observe possible side effects

## Patients and methods

### Study design

**Fig. 1:**
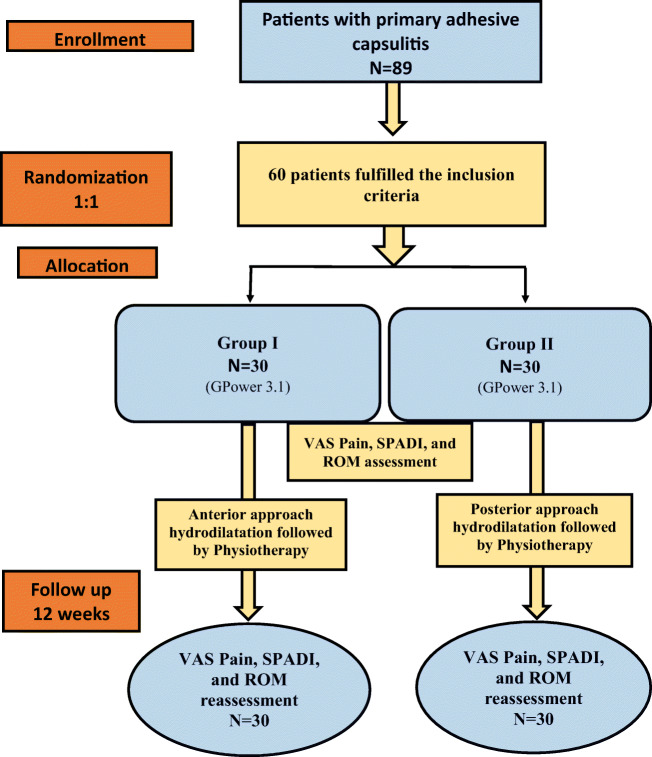
Flow chart

Prospective randomized controlled trial. See flow chart (Fig. 1).

### Patients

The study was carried out in new patients with idiopathic adhesive capsulitis of the shoulder. The patients were consecutively selected from the outpatient clinic of the physical medicine, rheumatology and rehabilitation departments, faculty of medicine, and Tanta university Hospitals, from January 2018 to June 2018. Patients who attended the clinic in the mentioned time frame and who fulfilled the inclusion criteria were subjected to randomization.

The inclusion criteria were patients aged 35 to 60 years, who suffered pain and stiffness in only one shoulder, for 1 to 6 months, and had restriction of passive motion, as measured with goniometer. The included patients were instructed to be off nonsteroidal anti-inflammatory drugs (NSAIDs), acetaminophen or opioid, and other pain killers at least 12 h before the procedure. Exclusion criteria were patients with previous trauma, neurological or endocrinal diseases like diabetes mellitus, and shoulder tumor; patients with arthritis; and people who had received an intra-articular shoulder injection within the last 6 months. Patients with tendon tear as shown by US (and MRI in case of doubt) were excluded.

All patients had a plain X-ray and diagnostic ultrasonography of the shoulder, done by a trained and expert rheumatologist, EULAR musculoskeletal ultrasound certified, to rule out any pathology that would exclude them from the study. MRI was only performed when indicated.

### Outcome measures

Clinical and functional assessment of the patients was done by assessment of pain in the shoulder by visual analogue scale (VAS) range 0–10 [14] and by a questionnaire measuring respectively shoulder pain and disability (SPADI) [15] (Pain VAS and SPADI are primary outcome measures).

The shoulder pain and disability index (SPADI) includes 5 questions for pain and 8 questions for disability, referring to various problems with their shoulder encountered over the last week. Each item is responded to by a visual analogue scale ranging from “no pain”/“no difficulty”, to “worst pain imaginable”/“so difficult required help”. Item scores for each section are averaged to produce separate subscale scores ranging from 0 to 100. A SPADI total score ranging from 0 (best) to 100 (worst) is then produced by averaging the two subscale scores [15].

The range of motion (ROM) regarding abduction, adduction, flexion, extension, external, and internal rotation were measured by investigators blinded for the injection approach (secondary outcome measure).

Clinical and functional assessments were done in all patients at baseline and 3 months after hydrodilatation followed by a guided physiotherapy exercise program. Possible side effects were administrated.

### Musculoskeletal ultrasound (MSKUS)

Ultrasonography was carried out for the shoulder, by using SAMSUNG MEDISON (UGEO H60) using linear, high-frequency probes (7.5–12 MHz). The imaging protocol for the shoulder evaluation followed the standard scans by EULAR anatomy images by Sono-anatomy Group—Barcelona University [16].

### Interventions

The enrolled patients were randomly divided by an external researcher into two groups of 32 patients each, according to the injection approach in the rotator interval. Randomization was carried out by the computer-generated block randomization. An independent external researcher without any contact with any of the patients carried out this randomization and allocation.

The ratio between group I patients who received ultrasound-guided hydrodilatation with corticosteroid, saline, and local anesthetic via posterior approach and group II patients who received ultrasound-guided hydrodilatation via anterior rotator interval approach was 1:1.

Complete information about the allocated group was given to the research assistants in sequential numbering in closed envelopes. Group allocation was completely blind to the principal investigator and outcome assessors. In addition, the statistician was blinded to participants till data analysis.

Both groups were injected ultrasound-guided by an expert radiologist with 1 ml methyl-prednisolone acetate (40 mg), 1 ml of 2% lidocaine, and 15 ml saline under strict aseptic conditions with total of 17 ml; both groups received the same injectable materials in the same amount.

Group I was treated through posterior approach; the patient was in semi-prone position. The affected shoulder is at the uppermost position, and the ipsilateral arm is placed over a pillow to maximize comfort and stability; the ultrasound transducer is positioned over the long axis of the myotendinous junction of the infraspinatus tendon just inferior to the scapular line to view the contours of the posterior glenoid rim, posterior glenoid labrum, and posterior portion of the humeral head; these structures must be viewed simultaneously on the ultrasound image as this is the correct injection spot. The injection needle is introduced at the skin surface just lateral to the transducer and in an oblique lateral to medial direction [17].

**Fig. 2:**
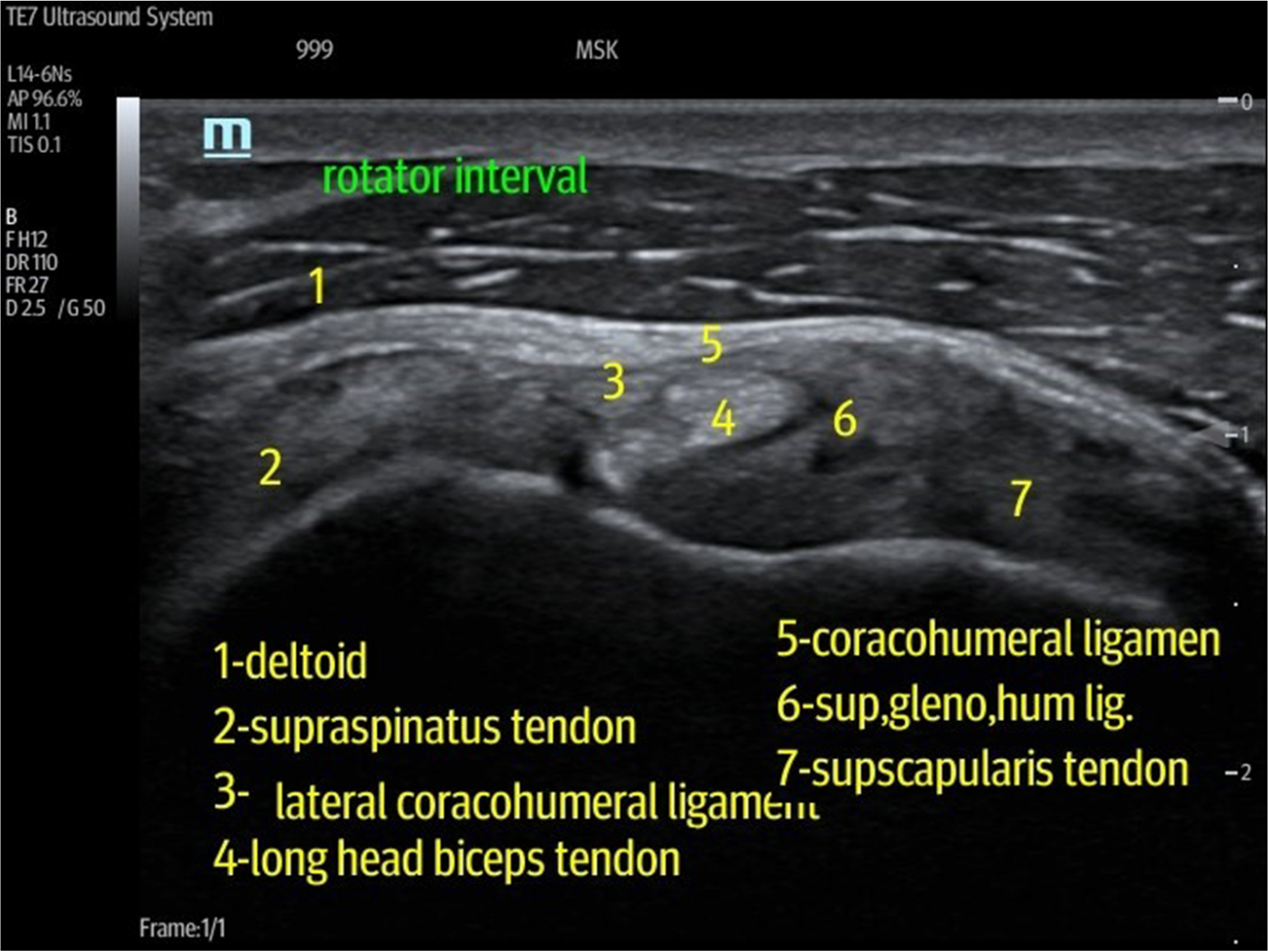
A transverse ultrasound image of the normal rotator interval with the biceps tendon (BT) at the center of the image void arrows. The superior glenohumeral ligament (SGHL) and lies anterior to the biceps tendon, and the coracohumeral ligament (CHL) lies superiorly, forming the roof of the interval. Lateral to BT lies the supraspinatus muscle (SS) and medially lies the subscapularis muscle (SUB). The blue arrow indicates the target point of the needle in RI technique

**Fig. 3:**
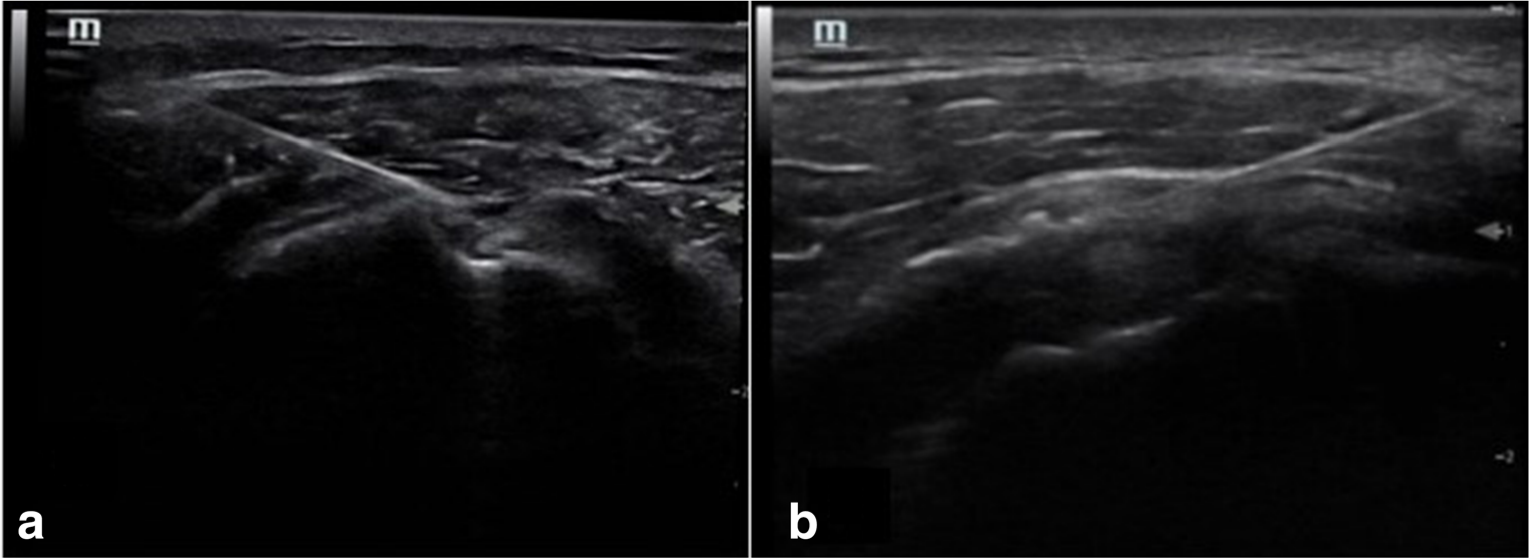
Ultrasound image of the transverse view of the right (**a**) and left (**b**) rotator interval. The needle tip lies between the coracohumeral ligament CHL above and biceps tendon sheath below

Group II was treated through anterior rotator interval approach; the patient lies supine or semi-supine with the affected shoulder closest to the radiologist. The shoulder is slightly extended, and the elbow flexed to facilitate visualization of the rotator interval anteriorly. The transducer is placed over the anterior shoulder, and a long-axis view of the rotator interval, with the biceps at the center of image and supraspinatus and subscapularis to either side, is obtained. The coracohumeral ligament is seen draped superiorly over the biceps tendon (Fig. 2). A 21-gauge needle is introduced into the rotator interval using an oblique path within the imaging plane of the transducer; from lateral to medial, the needle tip is imaged in real time throughout its passage from superficial to deep and is positioned in the biceps tendon sheath between the coracohumeral ligament above and biceps tendon below [18] (Fig. 3).

Both groups were given the same guided stretching and strengthening exercise program, every other day for 12 weeks after injection, by a trained musculoskeletal physiotherapist [19].

A total of 89 patients with primary adhesive capsulitis were recruited, out of which 60 patients could be included.

### Power analysis

A statistical power analysis was performed after sample size estimation, based on data from the current study (*N* = 60), comparing group I to group II. The effect size (ES) for VAS in this study was 0.81, considered to be large using Cohen’s (1988) criteria, with an alpha = 0.05 and sample size = 32 in every group; a post hoc power analysis was conducted with this effect size (G Power 3.1), and it is approximately (1-_β_) = 0.87. Thus, our power analysis for a sample size of 32 in every single group is adequate for the main objective of this study.

### Statistical analysis

Data were coded, tabulated, and analyzed using SPSS version 20 (Armonk, NY: IBM Corp). Qualitative data was expressed as numbers and percentages, and Chi-squared test (*χ*2) was applied to test the relationship between variables. The differences between baseline and follow-up for both groups were calculated, and a comparison between the differences between the groups was done. Quantitative data were expressed as mean and standard deviation (mean ± SD) and for noparametric variables, as median and interquartile range (IQR). The *t* test and Mann-Whitney U test were applied to test the relationship between independent variables. ANCOVA test was used to examine the differences in the mean values of dependent variables related to the effect of controlled independent variables. A *p* value of < 0.05 was considered as statistically significant.

### Ethics

The study conforms to the 1995 Helsinki declaration and was approved by the ethical committee of Tanta University Hospital. Written consent form was taken from all patients prior to their inclusion.

## Results

The mean age of the patients was 47.6 ± 3.5 years in group I and 45.4 ± 4.9 years in group II. Female participants accounted for 70% and 73.3% of group I and group II, respectively. All patients had primary adhesive capsulitis. Disease duration was 8.3 ± 2.68 months in group I and 9.1 ± 2.93 months in group II (*p* ≥ 0.05). In group I, a total of 15 right and 15 left shoulders were included, and in group II, these were 14 and 16, respectively (*p* ≥ 0.05) (Table 1).

**Table 1:** Demographic data of the patients with adhesive capsulitis subgroups according to the injection approach

Data	Adhesive capsulitis patients		Test	*p* value
	Group 1 (*n* = 32)	Group 2 (n = 32)		
Age (years)	47.6 ± 13.5	45.4 ± 4.9	0.83*	0.4
Sex				
Female Male	21 (70%) 9 (32%)	22 (73.3%) 8 (26.7%)	0.082**	0.77
Duration (months) range	8.3 ± 2.68 (5–18 months)	9.1 ± 2.93 (4–17 months)	1.3***	0.196
Affected shoulder				
Rt Lt	15 (50%) 15 (50%)	14 (46.6%) 16 (53.4%)	0.066**	0.79

*t test; **χ2 = chi square test; ***Mann-Whitney test

All baseline assessments (VAS pain, SPADI, and ROM regarding abduction, adduction, flexion, extension, external, and internal rotation) did not differ significantly between both groups (*p* ≥ 0.05) (Table 2).

**Table 2:** Comparison between posterior approach (group I) and anterior approach (group II) in patients with adhesive capsulitis regarding baseline pain, motion, and functional status before injection

Variable	Group I (n = 32)	Group II (n = 32)	Test	*p* value
VAS (pain) (0–10)	7.2 ± 9.61	7.23 ± 971	0.015**	0.98
Flexion (0–180)	99.35 ± 19.24	99 ± 19.13	0.01*	0.98
Extension (0–60)	43.16 ± 6.36	42.5 ± 7.28	0.44**	0.65
Abduction (0–180)	110.16 ± 21.87	108.83 ± 22.69	0. 34**	0.74
Adduction (0–45)	34.66 ± 7.03	32.35 ± 6.68	1.78**	0. 18
Internal rotation (0–90)	28.66 ± 9.53	26.5 ± 9.01	0.62**	0.53
External rotation(0–90)	40.66 ± 9.8	37.35 ± 10.4	0.84	0.4
SPADI (0–100)	89 ± 15.83	90.35 ± 15.19	0. 35*	0.74

*t test; **Mann-Whitney U test; VAS, visual analogue scale; SPADI, shoulder pain and disability index questionnaire

Group II had a significantly larger improvement regarding mean flexion, abduction, and external rotation, denoting improvement in ROM parameters (Table 3). Group II also showed significant lower mean VAS and SPADI values, denoting improvement of pain and functional status (Table 3). A nonsignificant difference was found between the two groups regarding the mean improvement in both extension and adduction. The improvement percentage in abduction was 3% for group I compared to 29% for group II. For external rotation, the improvement percentage was 13% for group I compared to 77% for group II. The improvement percentage in flexion was 3% for group I compared to 8% for group II.

**Table 3:** AVCOVA results and descriptive statistics of improvement in posterior approach (group I) and anterior approach (group II)

Variable	US-guided hydrodilatation with corticosteroid in shoulder adhesive capsulitis			
	*N* = 32	*N* = 32	ANCOVA test	*p* value
	Group I (adjusted mean)	Group II (adjusted mean)		
VAS Pain (0–10)	3.04	2.19	11.34	0.001
*ROM (°)*				
Flexion (0–180)	102.17	107.35	8.93	0.004
Extension (0–60)	43.77	43.82	0.004	0.95
Abduction (0–180)	143.81	153.68	8.63	0.005
Adduction (0–45)	34.22	31.6	4.81	0.34
Internal rotation (0–90)	28.26	28.46	0.15	0.69
External rotation (0–90)	45	61.35	52.1	< 0.001
SPADI (0–100)	74.95	38.97	152.8	< 0.001

VAS, visual analogue scale; SPADI, shoulder pain and disability index questionnaire

The difference between the values of pain, motion, and functional status before and after injections was calculated, and its mean was determined. When comparing the mean of the difference between the two groups, group II (anterior approach) showed a statistically significant higher mean improvement of VAS pain (*p* = 0.003), flexion, SPADI, abduction, and external rotation values before and after injections, compared to posterior approach (group I) (*p* < 0.001). The differences were also clinically relevant, especially for SPADI and external rotation. No significant difference was found between the two groups regarding adduction, extension, or internal rotation before and after injections (Table 4).

**Table 4:** Comparison between the two groups of adhesive capsulitis regarding the mean difference before and 3 months after injection regarding pain, motion, and functional status

Variable	Group I (mean ± SD)	Group II (mean ± SD)	Test	*p* value
VAS pain (0–10)	4.16 ± 1.01	5.03± 1.09	3*	0.003
*ROM (°)*				
Flexion (0–180)	3 ± 8.57	8.16 ± 3.82	4.26*	< 0.001
Extension (0–60)	0.28 ± 0.45	1 ± 0.72	0.5	0.61
Abduction (0–180)	35.84 ± 3.71	44.67 ± 13.77	4.15*	< 0.001
Adduction (0–45)	0.36 ± 1.58	0.2 ± 0.55	1.15*	0.26
Internal rotation (0–90)	1 ± 6.09	0.56 ± 1.54	1.62*	0.1
External rotation (0–90)	5.16 ± 4.04	22.83 ± 13.49	5.52*	< 0.001
SPADI (0–100)	14.66 ± 10.58	50.73 ± 11.79	12.46**	< 0.001

Posterior approach (Group I) and anterior approach (Group II)

*Mann-Whitney U- test; **t test; VAS, visual analogue scale; SPADI, shoulder pain and disability index questionnaire.

### Side effects

The procedure was well tolerated in both approaches; without complications, minor side effects were noticed after injection in 7 patients (3 in group I and 4 in group II) including transient local pain and facial flush, and pain in 3 patients (2 in group I and 1 in group II) was relieved with NSAIDs for 72 h.

## Discussion

Adhesive capsulitis is considered to be one of the most disabling painful shoulder conditions [3]. An inflamed subacromial and glenohumeral synovium, with coracohumeral ligament hypertrophy associated with fibrosis of the joint capsule, is considered the characteristic histopathological findings [20]. Intra-articular corticosteroid injection causes faster symptomatic relief than physiotherapy in adhesive capsulitis but with a short-term effect of less than 6 weeks [21]. Addition of a physiotherapy program following corticosteroid injections into glenohumeral joints was found to result in a statistical significant improvement [22].

The location of the corticosteroid injection in adhesive capsulitis influences the clinical response regarding pain and passive ROM [23]. In a recent study by Sun et al., ultrasound-guided injections of a mixture of 1 ml 40 mg/ml triamcinolone and 2 ml 2% lidocaine were applied for early frozen shoulder. A total of 77 patients (28 in the rotator interval group, 24 in the intra-articular posterior approach group, and 26 in the subacromial approach group) were analyzed at 4, 8, and 12 weeks after injection. The primary outcome was pain VAS with a scale of 10. Secondary outcomes included the constant score; the disability of arm, hand, and shoulder (DASH) score; and passive ROM, including flexion, abduction, external rotation with the arm at the side, and internal rotation with the arm at the side. The rotator interval injection was most effective, regarding primary and secondary outcomes (*p* < 0.001) followed by posterior approach and subacromial injection, even after 3 months [23]. These results are comparable with ours. In the current study, we found statistically significant improvements, after injection via rotator interval, regarding pain, ROM, and function in patients, rather than with the posterior approach. The difference with the study of Sun is that we applied hydrodilatation by adding saline to glucocorticosteroids and local anesthetics. To our knowledge, no direct study was done comparing intra-articular corticosteroids with and without hydrodistension.

**Fig. 4:**
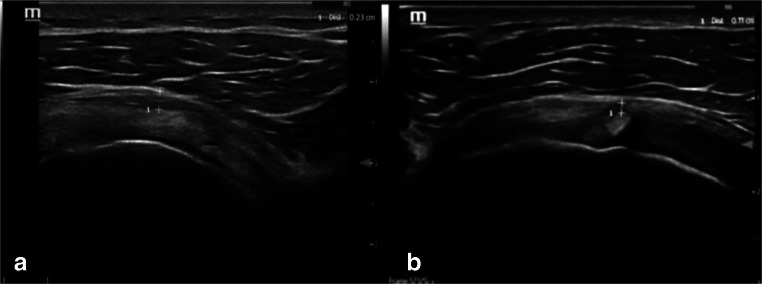
Ultrasound image of the of the rotator interval with thickened coracohumeral ligament (CHL) and anterior capsule in (**a**) patient with adhesive capsulitis in comparison to (**b**) healthy normal volunteer

**Fig. 5:**
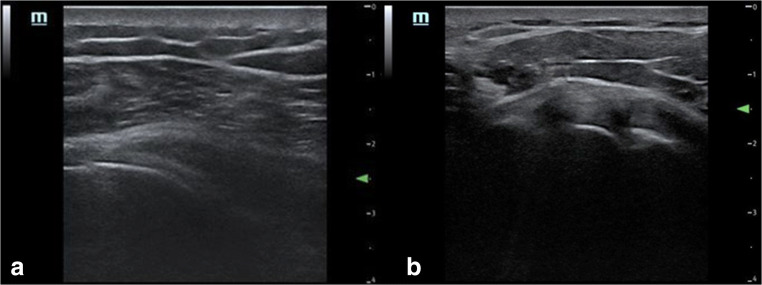
Ultrasound image of post injection via posterior approach (**a**) and transverse view post injection via rotator interval (**b**)

The predominant pathological finding in adhesive capsulitis is observed around the rotator interval and the anterior capsules [24] with a thickened coracohumeral ligament as shown by ultrasound [25] (Fig. 4), associated with obliteration of the subcoracoid fat on MRI [7]. The ultrasound image after injection via posterior approach (A) and transverse view post injection via rotator interval (B) is shown in Fig. 5.

The most commonly used approach of hydrodilatation is the posterior approach [17]. In the current study, we supposed that targeting the area of pathology could be of clinical significance. Treatment of concomitant bursitis can also result from fluid leakage into the adjacent subacromial bursa in some pathologic communication as well as adhesive capsulitis [18].

The present study has found a significant improvement in both anterior and posterior approach 3 months after hydrodilatation regarding VAS pain, external rotation, and SPADI. Only after anterior approach, improvement was observed regarding flexion and abduction. When comparing the two groups 3 months after hydrodilatation, there was statistically significant more improvement after the anterior rotator interval approach regarding VAS pain and SPADI, as well as regarding abduction and external rotation.

Bryant et al. studied the effectiveness of an ultrasound-guided hydrodistension (with 10 m lidocaine 1%, followed by 40 mg triamcinolone acetonide, and thereafter 20 ml of 0.9% NaCl), via posterior approach, followed by a guided exercise program, in adhesive capsulitis patients, in a primary care setting [26]. They found after 6 weeks and after 3 and 6 months a significant and continuing improvement on the SPADI scores, the Disability Arm Shoulder Hand (Quick DASH) scores, and clinical significant improvements of external rotation, flexion, and abduction movements compared to baseline. They did not compare the posterior approach with rotator interval approach [26]. The current study applied 15 ml saline with 1 ml lidocaine and 1 ml methyl-prednisolone acetate (40 mg) for hydrodilatation and had similar improvements regarding pain, abduction, external rotation of the shoulder joint, and SPADI, with posterior and anterior approach followed by physiotherapy program. The improvement in our study was also more significant in the rotator interval anterior approach. Pain and functional improvements have been found also in the previous hydrodilatation trials [13, 18]. Furthermore, short-term pain and disability improvements have been reported with intra-articular corticosteroid injection [21], which can be even more if the intra-articular injection is followed by physiotherapy [27].

Yoong et al. used hydrodilatation via rotator interval approach with a mixture of 10 ml of 1% lidocaine and 10 ml 0.5% bupivacaine and 1 ml steroid, total 21 ml [18].The pain (VAS 0–10) and function were assessed at 48 h and at 2 weeks and 4 months after injection by telephone survey. The Oxford Shoulder Questionnaire was done to assess shoulder symptoms prior and 4 months after shoulder dilatation [18]. There was no comparison group, but their findings were comparable with ours in the anterior RI approach. They found at 4 months, 19/22 (86%) of the patients had either complete (7/22) or good (12/22) improvement of their symptoms. The mean pain score improved from 8.4 to 3.1 at 48 h, to 2.1 at 2 weeks, and to 1.9 at 4 months; 20/22 (91%) of the patients had a lower pain score after 4 months. The Oxford shoulder score improved from 13.6 to 36.5 at 4 months (*p* < 0.05). In the current study, we had decided to use less lidocaine (1 ml) and 15 ml saline, as recent studies suggested that using large amounts of lidocaine had chondrotoxic effect [28].

In a recent review, Saltychev et al. found that in 12 studies, hydrodilatation combined with local corticosteroid showed to have a small size effect in patients with adhesive capsulitis with reduced pain reduction and improved ROM. No studies were found regarding anterior or posterior approach [29].

The mechanism of functional capacity and pain improvement via hydrodilatation of adhesive capsulitis is still unclear. Intrinsic and extrinsic factors could contribute to pathophysiology of adhesive capsulitis [30]. One of the possible intrinsic mechanisms that explain adhesive capsulitis is the increase of glycosaminoglycan concentration, which promotes myofibroblast activity; hydrodilatation may reverse the glycosaminoglycan action by the joint distension [30].

How can the better result of the anterior approach be explained?

The pathology in adhesive capsulitis starts in the rotator interval, and it includes soft tissue thickening in the rotator interval, which may encase the coracohumeral and superior glenohumeral ligaments, and soft tissue thickening adjacent to the biceps anchor [20]. Sectioning of rotator interval capsule and ligamentous structures increased passive ROM glenohumeral movements including flexion, extension, external rotation, and adduction in 80 cadaveric shoulders [31]. We hypothesized that the injection by anterior approach would increase the local corticosteroid concentration at the site of pathology, as it is a compact space, thus loosening adhesions via micro tear by increasing pressure. On the other hand, injecting into the posterior space, which is more roomy, would result in lesser capsular distension; although the injectable corticosteroid may somehow reach the rotator interval due to technical connection, it would do so in smaller amount. Further advantages of a targeted rotator interval (anterior) approach are that it can be used more efficiently particularly in contracted, irregular capsule, and biceps tenosynovitis that usually coexists with adhesive capsulitis [32]. In addition, it can be used in obese patients with subcutaneous fat due to better visualization through rotator interval than posterior glenohumeral recess. Facial pain expression can also be monitored using rotator interval anterior approach [18].

The subacromial space is often not connected with the joint space as shown in a study in rheumatoid arthritis patients [33]; this may possibly explain some of the differences between posterior and anterior approach. In adhesive capsulitis, there is some pathologic communication, and corticosteroid leakage through bursa can occur through anterior approach with beneficial effect [18].

The strength of this study is that it is the first prospective study comparing the effectiveness of shoulder ultrasound-guided hydrodilatation via rotator interval anteriorly versus posterior approach in adhesive capsulitis patients. The study was performed in the same setting between two comparable groups. The intervention was followed by a comprehensive exercise program by a physiotherapist for standardized monitoring and better outcome. The follow-up period was 3 months which is longer than many other studies.

This study has some limitation as there was no control group who received only corticosteroid injection with local anesthetic and no control group who received only physiotherapy and a group receiving only placebo injections. In our opinion, such control groups were not necessary to answer our research questions.

Till now, we do not have clear evidence what explains the improvement of adhesive capsulitis after hydrodilatation, whether it is related to capsule distension with hydrodilatation, corticosteroid, or the local anesthetic effect or the combination. Therefore, further longitudinal controlled randomized trials are needed to explain the underlying mechanisms.

## Conclusion

Ultrasound-guided anterior rotator interval hydrodilatation combined with local corticosteroid for adhesive capsulitis, followed by guided exercise, is clinically and functionally more effective than the conventional posterior approach.
